# Let’s Join the Lane: The Role of Infectious Diseases Physicians in Preventing Gun Violence

**DOI:** 10.1093/ofid/ofz026

**Published:** 2019-03-12

**Authors:** Crystal Zheng, David Mushatt

**Affiliations:** Section of Infectious Diseases, Tulane University School of Medicine, New Orleans, Louisiana

## Abstract

On November 7, 2018, the National Rifle Association (NRA) issued a tweet advising “self-important anti-gun doctors to stay in their lane.” The tweet has galvanized physicians to share their experiences with gun violence through the grassroots #ThisISOurLane campaign. Infectious diseases physicians are regularly called upon to manage complications such as infected wounds and osteomyelitis in gunshot victims. Yet, Infectious Diseases as a specialty has been poorly represented in the national dialogue on gun violence. Over 80 medical societies have endorsed statements on gun violence, including the American College of Physicians (ACP) and the American College of Cardiology; the Infectious Diseases Society of America has not. We argue that gun violence does affect the Infectious Diseases community and issue a call to action to engage in the conversation, advocate for our patients, and join with other medical societies in affirming a commitment to gun violence prevention.

## Teaser

Infectious diseases physicians are regularly called upon to manage complications of gunshot wounds, yet, as a specialty we have been poorly represented in the national dialogue on gun violence. We argue that gun violence is an infectious diseases issue and call on the IDSA to affirm a commitment to gun safety.

On November 7, 2018, the NRA issued a tweet advising “self-important anti-gun doctors to stay in their lane” [1].The tweet linked to an NRA article disparaging a position paper published by the ACP [2]. In its paper, the ACP reaffirms gun violence as a public health issue, provides evidence-based policy recommendations, and delineates the unique social responsibility physicians have to advocate for gun violence prevention [3]. In response to the NRA’s tweet, members of the medical community have taken to social media, sharing experiences with gun violence with the hash tag #ThisISOurLane. Many of the posts are by providers in fields on the “frontlines,” such as emergency medicine, critical care, and trauma surgery, often describing such harrowing experiences as holding pressure on a bleeding artery or breaking the news to parents that their child has died.

## GUN VIOLENCE IS AN INFECTIOUS DISEASES ISSUE

As infectious diseases physicians in Louisiana, the state with the third highest rate of firearm-related fatalities in the United States [4], we are all too familiar with the management of infections in gunshot victims. During the initial hospitalization, gunshot wounds can become infected, causing skin and soft tissue infections, as well as intra-abdominal, thoracic, and brain abscesses. Gunshot victims often have prolonged hospital and intensive care unit stays, predisposing them to nosocomial infections associated with multiple surgeries, ventilators, urinary catheters, and central lines.

One of the most challenging and heartbreaking consults we routinely receive is for a gunshot victim with infectious complications from spinal cord injury. Gunshot wounds are the third leading cause of spinal cord injury in the United States [5]. Long after the initial event, gunshot survivors with spinal cord injury face a lifetime of disability, marked by recurrent infections due to autonomic dysfunction, decreased airway clearance, sensory loss, and paraplegia. These patients are at increased risk for urinary tract infections, pneumonias, infected sacral ulcers, and chronic osteomyelitis [6]. Infection is the leading cause of death in this population [7]. The frequent use of antibiotics, high rate of readmissions, and prolonged hospitalizations lead to hospital-acquired infections and infections from multidrug-resistant organisms. The few studies that have attempted to quantify the costs associated with these infectious complications suggest a substantial economic burden. The cost of hospitalization for an infected pressure ulcer in gunshot wound victims was nearly $20 000 in one center [8], and the cost of hospitalization and outpatient antibiotics for osteomyelitis in another center reached approximately $60 000 [9]. The cumulative cost for each patient is multiplied when coupled with a readmission rate of 40% per year among spinal cord injury patients [10]. Moreover, a recent review of sacral pressure ulcers concluded that there is often futility in treating with long courses of intravenous antibiotics unless debridement and wound closure are performed, casting doubt on our ability to ever cure these infections [11].

The impact of gun violence does not stop with the victim. Family members who have lost loved ones experience psychological harm, affecting their ability to care for themselves. When one author’s human immunodeficiency virus (HIV)-positive patient was asked why he had been nonadherent with medications, he answered, “My son was murdered.” The collective trauma carried by a community so accustomed to gun violence can be felt across many different contexts in a city such as New Orleans.

## LET’S JOIN THE CONVERSATION

Despite the routine involvement of infectious diseases physicians in the care of gunshot victims, our field is not represented in the national conversation about gun violence. As of December 12, of thousands of #ThisISOurLane tweets, over 2000 contain the word “trauma.” We found only 5 tweets mentioning infections, 3 of which were penned by infectious diseases physicians. A search for articles about infectious complications of gunshot wounds in the 3 Infectious Diseases Society of America (IDSA) journals yielded just 5 articles in the last 10 years. Instead, the majority of articles on this topic are published in the surgical literature.

Gun violence is a multidisciplinary issue. In 2015, 8 medical professional societies jointly published a position paper advocating for gun control measures. These societies were the ACP, American College of Surgeons, American College of Obstetricians and Gynecologists, American Public Health Association, American Psychiatric Association, American Academy of Family Physicians, American Academy of Pediatrics, and the American College of Emergency Physicians [12]. More than 75 other medical societies have issued statements or endorsed positions on gun violence. These include both general medical groups such as the American Medical Association and specialist groups such as the American College of Cardiology (Table 1) [13–15]. The IDSA and the HIV Medical Association (HIVMA) have not released any such statement.

**Table 1.** National Medical Professional Societies Who Have Endorsed Position Statements on Gun Violence

Academic Pediatric Association	Association of Maternal & Child Health Programs
Alliance for Academic Internal Medicine	Association of Medical School Pediatric Department Chairs
American College of Medical Genetics and Gen omics	Association of Reproductive Health Professionals
American College of Physicians	Association of Schools and Programs of Public Health
American College of Preventive Medicine	Association of State and Territorial Health Officials
American Academy of Child and Adolescent Psychiatry	Association of Women’s Health, Obstetric and Neonatal Nurses
American Academy of Family Physicians	Commissioned Officers Association of the US Public Health Service, Inc.
American Academy of Neurology	Council of State and Territorial Epidemiologists
American Academy of Otolaryngology - Head and Neck Surgery	Council of Medical Specialty Societies
American Academy of Pediatrics	Doctors for America
American Academy of Physical Medicine and Rehabilitation	GLMA: Health Professionals Advancing LGBT Equality
American Art Therapy Association	Institute for Patient- and Family-Centered Care
American Association for Psychoanalysis in Clinical Social Work	International Association of Forensic Nurses
American Association of Colleges of Nursing	National Alliance to Advance Adolescent Health
American College of Cardiology	National Association for Children’s Behavioral Health
American College of Chest Physicians	National Association of Community Health Centers
American College of Emergency Physicians	National Association of County and City Health Officials
American College of Nurse-Midwives	National Association of Pediatric Nurse Practitioners
American College of Obstetricians and Gynecologists	National Association of School Nurses
American College of Occupational and Environmental Medicine	National Association of State EMS officials
American College of Surgeons	National Black Nurses Association
American Counseling Association	National Board of Medical Examiners
American Geriatrics Society	National Hispanic Medical Association
American Medical Association	National Medical Association
American Medical Student Association	National Network of Public Health Institutes
American Medical Women’s Association	National Physicians Alliance
American Nurses Association	National Register of Health Service Psychologists
American Osteopathic Association	North American Society for Pediatric Gastroenterology, Hepatology and Nutrition
American Pediatric Society	Patient-Centered Primary Care Collaborative
American Pediatric Surgical Association	Pediatric Policy Council
American Psychiatric Association	Physicians for Social Responsibility
American Psychoanalytic Association	Prevention Institute
American Psychological Association	Public Health Institute
American Public Health Association	Society for Adolescent Health and Medicine
American Society for Clinical Pathology	Society for Behavioral Medicine
American Society of Hematology	Society for Pediatric Research
American Society of Nuclear Cardiology	Society for Public Health Education
American Thoracic Society	Society of Critical Care Medicine
Association for Ambulatory Behavioral Healthcare	Society of General Internal Medicine
Association of American Medical Colleges	Society of Thoracic Surgeons
Association of Black Cardiologists	Urgent Care Association of America
Association of Chiefs and Leaders of General Internal Medicine	

Infectious diseases physicians are no strangers to advocacy. Since the early days of the HIV epidemic, the field has championed for funding for HIV research, access to medications, and destigmatization of patients. Continuing in that tradition, both the IDSA and HIVMA websites include sections dedicated to “Policy & Advocacy,” which detail policy statements and outline specific actions members can take on topics ranging from antibiotic resistance to marijuana.

## CALL TO ACTION

Source control is a foundational principle of infectious diseases. Although we can, and we will, treat gunshot victims each time they come through the door, the role of our antibiotic interventions is limited. If we consider gun violence to be a public health epidemic, as many medical societies do and the number of people impacted suggests we must, we should apply the concepts of source control, prevention, and harm reduction, concepts very familiar to the infectious diseases physician, to reduce its impact. Translated into policy, specific measures we as infectious disease physicians can advocate for include those endorsed in the ACP position paper, such as regulations on the purchase of firearms, education on firearm safety, and built-in safety devices.

## CONCLUSIONS

This is a call to action to our infectious diseases colleagues. To advocate for gun violence prevention, we need to first acknowledge that it “is” within the scope of infectious diseases. Let’s add our voices to the conversation. Let’s publish more research about infections and gun violence. Let’s advocate for our patients. Let’s get on par with other medical societies. Let’s let everyone know #ThisISOurLane.

We, the undersigned members of the Infectious Diseases Section of the Tulane University School of Medicine, endorse the ideas expressed in this article:

Crystal Zheng, MD, MAPP ^1^

Yardley Brice, DO^1^

John Dwyer, DO^1^

April McDougal, DO, MS^1^

Erin Boswell, MD, MSc^1^

Allison Cormier, MD^1^

Dahlene Fusco, MD, PhD^1^

Jason Halperin, MD^1, 2^

Nadine M. Harris, MD^1, 2^

Jo-Ann Jose, MD, MPH&TM^1, 2^

Alfred Luk, MD^1^

Jeffrey Percak, MD^1^

John Schieffelin, MD, MSPH^1^

Edwin Swiatlo, MD, PhD^1^

Nicholas Van Sickels, MD^ 1, 2^

Kyle Widmer, MD^ 1^

Philip A. Yeon, MD, MPH&TM^ 1^

David Mushatt, MD, MPH&TM, FIDSA, FACP^1^

^1^Section of Infectious Diseases, Tulane University School of Medicine, New Orleans, Louisiana

^2^CrescentCare, New Orleans, Louisiana

## References

[1] National Rifle Association of America. [NRA]. Someone should tell self-important anti-gun doctors to stay in their lane. Half of the articles in Annals of Internal Medicine are pushing for gun control. Most upsetting, however, the medical community seems to have consulted NO ONE but themselves [Tweet] 2018 Available at: https://twitter.com/NRA/status/1060256567914909702 Accessed 12 December 2018.

[2] National Rifle Association of America. Surprise: physician group rehashes same tired gun control policies Available at: https://www.nraila.org/articles/20181102/surprise-physician-group-rehashes-same-tired-gun-control-policies Accessed 12 December 2018.

[3] Butkus R, Doherty R, Bornstein SS, et al Reducing firearm injuries and deaths in the United States: a position paper from the American College of Physicians. Ann Intern Med 2018; 169:704–7. 30383132 10.7326/M18-1530

[4] Centers for Disease Control and Prevention. Firearm mortality by state Available at: https://www.cdc.gov/nchs/pressroom/sosmap/firearm_mortality/firearm.htm Accessed 12 December 2018.

[5] Jain NB, Ayers GD, Peterson EN, et al. Traumatic spinal cord injury in the United States, 1993-2012. JAMA 2015; 313:2236–43. 26057284 10.1001/jama.2015.6250 PMC4712685

[6] Garcia-Arguello LY, O’Horo JC, Farrell A, et al Infections in the spinal cord-injured population: a systematic review. Spinal Cord 2017; 55:526–34. 27922625 10.1038/sc.2016.173

[7] National Spinal Cord Injury Statistical Center. 2017 Annual Statistical Report for the Spinal Cord Injury Model Systems Public Version. Birmingham, Alabama: University of Alabama at Birmingham Available at: https://www.nscisc.uab.edu Accessed 12 December 2018.

[8] Chopra T, Marchaim D, Awali RA, et al Risk factors and acute in-hospital costs for infected pressure ulcers among gunshot-spinal cord injury victims in southeastern Michigan. Am J Infect Control 2016; 44:315–9. 26619947 10.1016/j.ajic.2015.10.002

[9] Hirshberg J, Rees RS, Marchant B, Dean S Osteomyelitis related to pressure ulcers: the cost of neglect. Adv Skin Wound Care 2000; 13:25–9. 11061707

[10] Savic G, Short DJ, Weitzenkamp D, et al Hospital readmissions in people with chronic spinal cord injury. Spinal Cord 2000; 38:371–7. 10889566 10.1038/sj.sc.3101019

[11] Wong D, Holtom P, Spellberg B Osteomyelitis complicating sacral pressure ulcers: whether or not to treat with antibiotic therapy. Clin Infect Dis 2019; 68:338–42. 29986022 10.1093/cid/ciy559 PMC6594415

[12] Weinberger SE, Hoyt DB, Lawrence HC 3rd, et al Firearm-related injury and death in the United States: a call to action from 8 health professional organizations and the American Bar Association. Ann Intern Med 2015; 162:513–6. 25706470 10.7326/M15-0337

[13] American College of Cardiology [ACCinTouch]. Statement from the American College of Cardiology on Gun Violence @ACPinternists @AmerMedicalAssn #thisisourlane [Tweet] 2018 Available at: https://twitter.com/ACCinTouch/status/1060931393889419264 Accessed 12 December 2018.

[14] American College of Physicians. Firearm-related injury and death in the United States: a call to action from over 50 supportive organizations and the American Bar Association Available at: https://www.acponline.org/system/files/documents/advocacy/where_we_stand/assets/firearms-policy-endorsing-organizations-11-4-15.pdf Accessed 12 December 2018.

[15] American Academy of Family Physicians. Joint letter to House urging bipartisan gun safety policies Available at: https://www.aafp.org/dam/AAFP/documents/advocacy/prevention/safety/LT-House-ParklandGVP-022218.pdf Accessed 12 December 2018.

